# The integration of mHealth technologies in telemedicine during the COVID-19 era: A cross-sectional study

**DOI:** 10.1371/journal.pone.0264436

**Published:** 2022-02-24

**Authors:** Abdul Rahman Taha, Mustafa Shehadeh, Ali Alshehhi, Tariq Altamimi, Emma Housser, Mecit Can Emre Simsekler, Buthaina Alfalasi, Shammah Al Memari, Farida Al Hosani, Yousif Al Zaabi, Shereena Almazroui, Hamed Alhashemi, Noora Alhajri

**Affiliations:** 1 College of Medicine and Health Sciences, Khalifa University, Abu Dhabi, UAE; 2 College of Engineering, Khalifa University, Abu Dhabi, UAE; 3 Department of Family Medicine, Zayed Military Hospital, Abu Dhabi, UAE; 4 Abu Dhabi Public Health Center (ADPHC), Abu Dhabi, UAE; 5 Department of Health (DoH), Abu Dhabi, UAE; 6 Department of Medicine, Sheikh Shakhbout Medical City (SSMC), Abu Dhabi, UAE; Kasturba Medical College Mangalore / Manipal Academy of Higher Education, INDIA

## Abstract

Telemedicine is a rapidly expanding field of medicine and an alternative method for delivering quality medical care to patients’ fingertips. With the COVID-19 pandemic, there has been an increase in the use of telemedicine to connect patients and healthcare providers, which has been made possible by mobile health (mHealth) applications. The goal of this study was to compare the satisfaction of patients with telemedicine among mHealth users and non-users. This was a survey-based study that included outpatients from Abu Dhabi. The association between patient satisfaction with telemedicine and use of mHealth technologies was described using regression models. This study included a total of 515 completed responses. The use of mHealth application was significantly associated with ease of booking telemedicine appointments (OR 2.61, 95% CI 1.63–4.18; P < .001), perception of similarity of quality of care between telemedicine consultations and in-person visits (OR 1.81, 95% CI 1.26–2.61; P = .001), and preference for using telemedicine applications over in-person visits during the COVID-19 pandemic (OR 1.74, 95% CI 1.12–2.72; P = .015). Our study results support that the use of mHealth applications is associated with increased patient satisfaction with telemedicine appointments.

## 1. Introduction

Over the last decade, the digital revolution led by smartphones has made headway in all aspects of life, including healthcare [1]. This revolution began with the exponential rise of the smartphone, along with its counterparts in tablet technology and wearables such as smart watches. These have evolved to cover applications including wearable sleep technology [2], applications that record patient’s electrocardiogram (ECG) [3] and detect falls in geriatric patients [4]. Such applications, coupled with high-speed internet connection, have made it possible to effectively connect patients to their doctors via text, audio, and video within a matter of seconds, which catalyzed the emergence of telemedicine [5]. Traditionally, telemedicine was delivered via networks of dedicated sites at clinics and hospitals where patients would attend their virtual appointments with a clinician at a site closest to their home with clinicians also joining via their closest clinic or hospital [6,7].

Telemedicine is defined by the World Health Organization as “the use of electronic communications and information technologies to provide clinical services when participants are at different locations’’ and “a tool that can be used by health providers to extend the traditional practice of medicine outside the walls of the typical medical practice” [8]. The drive towards telemedicine began many years ago, but with the surge of the COVID-19 pandemic, the telemedicine initiative was accelerated such that it has been estimated that within the first two months of the pandemic, utilization of telehealth had increased by 78 times as opposed to the pre-COVID-19 baseline [9]. The use of telemedicine during the COVID-19 pandemic has proved to be advantageous to relieve the load building on healthcare facilities and reserve the hospital capacity for high-risk patients [10]. Additionally, this has reduced risk of infection by close contact [11], eliminated some barriers for patients with functional impairments [12], and provided easier access for patients living in rural areas considered health deserts [13,14]. The United Arab Emirates (UAE) is one such country that has recently followed suit with the introduction of the “Remote Healthcare Platform” in conjunction with the Department of Health (DoH) within the Emirate of Abu Dhabi. This initiative aimed to “curb the spread of the COVID-19 and offer alternative digital solutions to ensure the safety and well-being of the society” [15]. Through this platform, patients can book and manage appointments and in turn attend remote consults via video, audio, or text, as well as request prescriptions and receive preliminary diagnoses from the convenience of a centralized application with a 2-way messaging system on their smartphones [16], in contrast to the traditional telemedicine consultation, which is typically delivered via phone or, less frequently, computer-based audio or video calls [17]. This platform was envisioned to meet the needs of, and provide, “an opportunity for all the members of the community to get access to safe, convenient healthcare at the comfort of their homes” [16]. This focused on “patients with chronic illnesses, the elderly and others in need of prescriptions to have their needs met without the need to visit hospitals where they are more susceptible to infection, while also reducing the duration of their medical treatment journey and enhancing their experience” [15]. Because many of these health systems were implementing telemedicine mobile applications for the first time, it was unclear whether patient acceptance of this new technology for delivering healthcare services differed or whether satisfaction differed between mHealth users and non-users. Davis et al. used the technology acceptance model (TAM) to describe the acceptance of new technology for the first time in 1989 [18]. This model consists of two major constructs: 1) perceived usefulness (PU) and 2) perceived ease of use of new technology (PEOU). TAM can assist in better understanding patients’ attitudes toward receiving clinical care via new online innovations such as telemedicine mobile applications. The Centers for Medicare and Medicaid Services (CMS) defined patient satisfaction as "the patient perspective of healthcare services that can be used as an objective metric to contrast quality of healthcare services" [19]. In all aspects of healthcare, patient satisfaction is gaining increasing attention [20]. It is an important metric that is used frequently to evaluate the quality of healthcare services. Therefore, while patients’ satisfaction is a proxy, it is an efficient way to assess the quality of healthcare services, according to the report published by the United States Healthcare Effectiveness Data and Information Systems (HEDIS) [21,22].

While the implication of telemedicine mobile applications still faces certain challenges and barriers [23–26], adoption of these technologies has been on the rise [9]. Our sister study has previously reported on physician’s attitude and confidence towards telemedicine in treating acute conditions, providing patients with health education and enhancing the patient-physician rapport [27]. This study on the other hand, aimed to explore the impact of using a mobile application to manage and attend patient-physician consultations on the overall satisfaction of the patients using telemedicine in Abu Dhabi. We hypothesize that using the “Remote Care” application can increase overall patient satisfaction using telemedicine in Abu Dhabi, particularly during the course of a pandemic.

## 2. Materials and methods

### 2.1. Study design

This was a survey-based study on outpatients who used telemedicine services in Abu Dhabi during the COVID-19 pandemic in December 2020. An online survey was used to collect data, which was sent via an internal SMS system [21]. Ethics approval was obtained from the institutional review board of Khalifa University (protocol# H21-006-2020) and of the Abu Dhabi COVID-19 Research Committee of the Department of Health in Abu Dhabi (reference# DOH/CVDC/2020/1747). Surveys were administered through the Department of Health and SEHA, these being the major health authorities in Abu Dhabi. The institutional review board or ethics committee at each participating institution approved the study protocol and survey. Electronic written consent was waived for this data-only study owing to the deidentified nature of this survey.

#### 2.1.1 Subject selection, inclusion, and exclusion criteria

This study used volunteer sampling as its sampling method. The calculated sample size to achieve 80% power was 426 participants, with a 20% non-response rate adjustment. The online survey link was primarily distributed through the internal SMS system of the Department of Health (DOH) and Abu Dhabi Health Company (SEHA), two major health regulatory authorities in Abu Dhabi that maintained a registry of patients who visited outpatient facilities (hospital OPD and community clinics) during the COVID-19 pandemic. Participants must be at least 18 years old and have completed a telemedicine consultation in an outpatient setting during the COVID-19 pandemic, which runs from March to December 2020. Patients who had never used telemedicine services during the COVID-19 pandemic were excluded.

#### 2.1.2 Survey development, piloting, and data collection

The survey was developed by a team of physicians who frequently consulted patients using telemedicine services during the COVID-19 pandemic and was available in English and Arabic language. The survey consisted of demographic characteristics and five Likert scale questions. Two main outcomes were examined in this survey “acceptance of telemedicine” and “satisfaction with telemedicine”. The first outcome “acceptance of telemedicine” was examined through two main constructs: a) perceived usefulness (PU) of telemedicine and the second construct: b) perceived ease of use (PEOU) of telemedicine. A pilot study with 30 patients was conducted to determine whether the questions were understandable, appropriate, well defined, and understood consistently. The study investigators also assessed the patient’s information statement for appropriateness and comprehension [27]. The online survey tool was created using the Microsoft Forms platform (Microsoft Corporation 2018, Redmond, WA). The survey was conducted over a two-week period (December 2nd-December 16th, 2020), with the initial invitation sent in the first week and a reminder invitation sent in the second week to increase subject recruitment.

### 2.2. Study variables and outcomes

Sociodemographic factors including age, sex, education level, marital status, past experience with telemedicine, distance to the healthcare facility and the use of mHealth “Remote Care” application were all self-reported by survey respondents. We compared patient satisfaction with telemedicine services among users of the telemedicine mHealth application versus non-users who used the traditional telemedicine system. The mHealth “Remote Care” application provided a real-time virtual encounter where patients and physicians can communicate using video conferencing software built within a customized patient portal with a secured two-way patient-to-provider email exchange in addition to an automated schedule of the provider’s telemedicine appointment availability. While the traditional telemedicine system refers to booking the remote consultation through the hospitals’ operating system, which may be slow, and may not necessarily provide the secured two-way email or message exchange between the patient and the provider. The level of agreement with multiple quality-related constructs has been used to measure telemedicine quality, which has been defined as patients’ satisfaction with the telemedicine services. Patient satisfaction was defined as the person’s overall perceptions of quality of healthcare services. We used a multi-item approach to evaluate patients satisfaction with the quality of telemedicine services by rating the following statements using a 5-point Likert scale: 1) It was easy to book telemedicine appointment 2) The doctor could hear me clearly during the telemedicine consultation 3) I could hear the doctor clearly using the telemedicine system 4) the care provided through the telemedicine system is the same as in-person consultation 5) I prefer to use telemedicine over in-person visit during and after the COVID-19 pandemic.

### 2.3. Statistical analysis

In this study, we compared the differences in patient’s satisfaction with telemedicine among the “Remote Care” mHealth users versus non-users by assessing five quality aspects of the telemedicine services provided. Descriptive statistics characterizing the survey respondents were reported as frequency and percentages for all outcomes. To compare responses to survey questions between DoH “Remote Care” application users and non-users, we performed Chi-square statistical tests at a significance level of 0.05. At this stage, we have checked the distribution of the responses in 5-point Likert scale questions and found limited observations, particularly towards the extreme negative and positive ends of the scale (i.e., strongly disagree and strongly agree). We combined strongly agree and agree under positive direction, and strongly disagree and disagree under negative direction, due to limited observations in such cases, as these statements were determined to involve the same attitude continuum toward the question. Therefore, the outcomes were collapsed into ‘disagreement’, ‘neutral’ and ‘agreement’ as carried out in similar studies [27]. Furthermore, we used ordered logistic regression analyses to investigate the association between the types of application users and outcome variables (perceived quality and patient satisfaction), adjusting for confounding factors such as socio-demographic characteristics. Considering the variance inflation factor (VIF) diagnostic, we adopted a forced-entry approach to prevent unreliable estimates of coefficients and odds ratios due to high correlations among predictor variables. Results showed no multicollinearity as a concern in the final models (VIF = 1.1). Regression results were reported as odds ratios (ORs) with 95% confidence intervals (CI) and p < 0.05 demonstrating statistical significance. Statistical analyses were performed using STATA 16.1 (Stata Corp LLC, USA).

Following the statistical analysis, the perception of remote health application users was investigated by asking four questions. These questions aimed to capture significant insights on the overall satisfaction, information accuracy, usability, and whether users would recommend the application to others. The proportions of patients who agreed, disagreed, or were neutral for all these statements are then demonstrated in a chart.

## 3. Results

A total of 515 patients completed the survey, of which 169 (32.82%) used the “Remote Care” application for their telemedicine consultation, while 346 (67.18%) used the traditional telemedicine system. The socio-demographic characteristics of the two groups are presented and compared in **Table 1**.

**Table 1:** Patient socio-demographic characteristics and descriptive statistics by “Remote Care” application user type.

Variables	Non-mHealth users, n (%) 346 (67.18)	mHealth users, n (%) 169 (32.82)	Total, n (%) 515 (100.00)	P value
Sex				0.04
Male	143 (41.33)	86 (50.89)	229 (44.47)	
Female	203 (58.67)	83 (49.11)	286 (55.53)	
Age range, y				0.382
<39	152 (43.93)	63 (37.28)	215 (41.75)	
40–49	93 (26.88)	56 (33.14)	149 (28.93)	
50–59	64 (18.50)	34 (20.12)	98 (19.03)	
60+	37 (10.69)	16 (9.46)	53 (10.29)	
Education				0.424
High school or eqv.	115 (33.24)	66 (39.05)	181 (35.15)	
High degree or eqv.	177 (51.16)	78 (46.16)	255 (49.51)	
Higher degree or eqv.	54 (15.61)	25 (14.79)	79 (15.34)	
Marital Status				0.196
Single	65 (18.79)	23 (13.61)	88 (17.09)	
Married	251 (72.54)	135 (79.88)	386 (74.95)	
Others (Widowed And Divorced)	30 (8.67)	11 (6.51)	41 (7.96)	
Past experience with Telemedicine				0.072
Never Used	215 (62.14)	91 (53.85)	306 (59.42)	
Used	131 (37.86)	78 (46.15)	209 (40.58)	
Employment				0.04
Employed	227 (65.61)	126 (74.56)	353 (68.54)	
Unemployed	119 (34.39)	43 (25.44)	162 (31.46)	
Distance to health center				0.243
< 30 min	267 (77.17)	138 (81.66)	405 (78.64)	
> 30 min	79 (22.83)	31 (18.34)	110 (21.36)	

### 3.1. Socio-demographic differences

When comparing users of the non-remote application for telemedicine consultations, those who used the “Remote Care” application were majority males (50.89% vs 41.33%, respectively; P = .04), middle aged (40–49 years: 33.14% vs 26.88%, 50–59 years: 20.12% vs 18.50%, P = .382), more likely to have a high school degree or equivalent (39.05% vs. 33.24%, P = .424), married (79.88% vs. 72.54%, P = .196), employed (74.56% vs. 65.61%, P = 0.04), lived within 30 minutes of a health center (81.66% vs. 77.17%, P = .243), and had a previous experience with telemedicine (46.15% vs. 37.86%, P = .072). On the other hand, those who used the non-remote application were majority younger females, with a high or higher degree or equivalent, more likely than their counterparts to be single or of other marital status, unemployed, have never used telemedicine and live more than 30 minutes away from a health center.

### 3.2. Perceived usefulness (PU) and ease of use (PEOU) of telemedicine mobile applications

Patient satisfaction with the telemedicine system via the “Remote Care” application was assessed using a multi-item approach system. The number of patients who agreed, disagreed or answered neutral about the statements are shown in **Table 2**. Overall, patients who used the “Remote Care” application were more likely to agree that scheduling telemedicine appointments was easy as opposed to patients who did not use the “Remote Care” application (83.43% vs 64.16%, P < .001). Additionally, “Remote Care” application users were more likely to indicate that the care provided was similar between telemedicine and in-person consultation (59.76% vs 44.80%, P = .003), and preferred to use telemedicine over in-person visits during COVID-19 (79.88% vs 69.08%, P = .026). However, there was no significant difference in the audio clarity during the telemedicine consultations between the groups, specifically in terms of being heard by the physician (84.62% vs 90.64%, P = .443), and in hearing the physician during the remote consultation (85.21% vs 82.37%, P = .672).

**Table 2:** Comparison of survey responses on the perceived usefulness and ease of use of telemedicine services provided by mobile application users versus non-users.

Outcome Variables	m-Health users, n (%) 346 (67.18)	non m-Health users, n (%) 169 (32.82)	Total, n (%) 515 (100.00)	P value
It was easy to book telemedicine appointment				<0.001
Disagree & Strongly Disagree	39 (11.27)	12 (7.10)	51 (9.90)	
Neutral	85 (24.57)	16 (9.47)	101 (19.61)	
Agree & Strongly Agree	222 (64.16)	141 (83.43)	363 (70.49)	
The doctor could hear me clearly during the telemedicine consultation				0.448
Disagree & Strongly Disagree	20 (5.78)	6 (3.55)	26 (5.05)	
Neutral	47 (13.58)	20 (11.83)	67 (13.01)	
Agree & Strongly Agree	279 (80.64)	143 (84.62)	422 (81.94)	
I could hear the doctor clearly using the telemedicine system				0.672
Disagree & Strongly Disagree	17 (4.91)	8 (4.73)	25 (4.85)	
Neutral	44 (12.72)	17 (10.06)	61 (11.84)	
Agree & Strongly Agree	285 (82.37)	144 (85.21)	429 (83.31)	
The care provided through the telemedicine system is as good as the in-person consultation				0.003
Disagree & Strongly Disagree	92 (26.59)	26 (15.38)	118 (22.91)	
Neutral	99 (28.61)	42 (24.85)	141 (27.38)	
Agree & Strongly Agree	155 (44.80)	101 (59.76)	256 (49.71)	
I prefer to use telemedicine over the in-person visit during and after the COVID-19 pandemic				0.026
Disagree & Strongly Disagree	50 (14.45)	13 (7.69)	63 (12.23)	
Neutral	57 (16.47)	21 (12.43)	78 (15.15)	
Agree & Strongly Agree	239 (69.08)	135 (79.88)	374 (72.62)	

### 3.3. Multivariate analysis

In the multivariate model analysis, patients who used the “Remote Care” application were about three times as likely to report an increased ease of booking telemedicine appointments (OR 2.61, 95% CI 1.63–4.18; P < .001), almost two times more likely to have a perception of similarity between the quality of care in telemedicine and in-person consultations (OR 1.81, 95% CI 1.26–2.61; P = .001), and almost two times more likely to prefer using telemedicine over in-person visits during the COVID-19 pandemic (OR 1.74, 95% CI 1.12–2.72; P = .015) (**Table 3**). There was no significant difference between “Remote Care” users and non-users in hearing the physician or being heard by the physician during the telemedicine consultation. Additionally, there was no significant difference between other sociodemographic characteristics and other variable outcomes.

**Table 3:** Adjusted multivariate models for satisfaction with the telemedicine system.

Variables	Ease of booking telemedicine appointment		The doctor can hear me clearly during the consultation		I can hear the doctor clearly during the consultation		The care provided through telemedicine is as good as the in-person consultation		Prefer to use telemedicine over the in-person consultation	
	OR (95% CI)	P value	OR (95% CI)	P value	OR (95% CI)	P value	OR (95% CI)	P value	OR (95% CI)	P value
mHealth users user (vs. non-user)	2.61 (1.63–4.18)	<0.001	1.34 (0.81–2.23)	0.259	1.25 (0.74–2.11)	0.407	1.81 (1.26–2.61)	0.001	1.74 (1.12–2.72)	0.015
Sex female (vs. male)	1.15 (0.75–1.75)	0.520	1.32 (0.80–2.17)	0.274	1.19 (0.71–1.99)	0.504	1.15 (0.80–1.65)	0.445	1.14 (0.75–1.74)	0.538
Age Range, Y										
<39 vs. 60+	0.80 (0.40–1.60)	0.526	0.93 (0.42–2.07)	0.868	1.38 (0.64–2.99)	0.408	1.65 (0.91–3.00)	0.099	0.78 (0.38–1.61)	0.506
40–49 vs. 60+	0.83 (0.40–1.72)	0.622	1.22 (0.52–2.83)	0.647	1.99 (0.87–4.52)	0.102	1.71 (0.91–3.20)	0.093	0.89 (0.41–1.90)	0.760
50–59 vs. 60+	1.60 (0.73–3.48)	0.238	1.55 (0.62–3.88)	0.353	4.46 (1.59–12.49)	0.004	1.77 (0.93–3.39)	0.084	1.14 (0.51–2.54)	0.747
Education										
high degree vs. high school	0.85 (0.54–1.33)	0.471	0.69 (0.40–1.18)	0.173	0.89 (0.51–1.54)	0.668	0.70 (0.47–1.04)	0.075	0.93 (0.59–1.47)	0.761
higher degree vs. high school	0.99 (0.52–1.86)	0.971	0.84 (0.40–1.77)	0.647	1.32 (0.59–2.94)	0.494	0.61 (0.36–1.05)	0.073	0.87 (0.47–1.62)	0.660
Marital Status										
Married vs. single	0.71 (0.41–1.23)	0.223	0.62 (0.31–1.24)	0.176	0.52 (0.25–1.09)	0.084	1.18 (0.74–1.88)	0.477	1.05 (0.62–1.78)	0.857
Others vs. single	0.59 (0.25–1.40)	0.231	0.70 (0.23–2.16)	0.536	0.70 (0.21–2.40)	0.576	0.89 (0.42–1.86)	0.751	0.68 (0.28–1.63)	0.390
Past experience with TM Used vs. never used	1.14 (0.76–1.70)	0.531	1.10 (0.69–1.77)	0.680	0.98 (0.60–1.59)	0.922	0.99 (0.70–1.39)	0.953	1.18 (0.79–1.78)	0.418
Employment employed vs. unemployed	1.30 (0.81–2.08)	0.282	0.87 (0.49–1.56)	0.642	0.63 (0.34–1.18)	0.152	0.92 (0.61–1.40)	0.710	1.13 (0.70–1.83)	0.619
Distance to health center > 30 min vs. < 30 min	0.73 (0.46–1.14)	0.161	0.63 (0.38–1.06)	0.080	0.63 (0.37–1.06)	0.080	0.94 (0.63–1.42)	0.782	0.79 (0.50–1.25)	0.314

### 3.4. Satisfaction with mHealth in telemedicine

**Fig 1:**
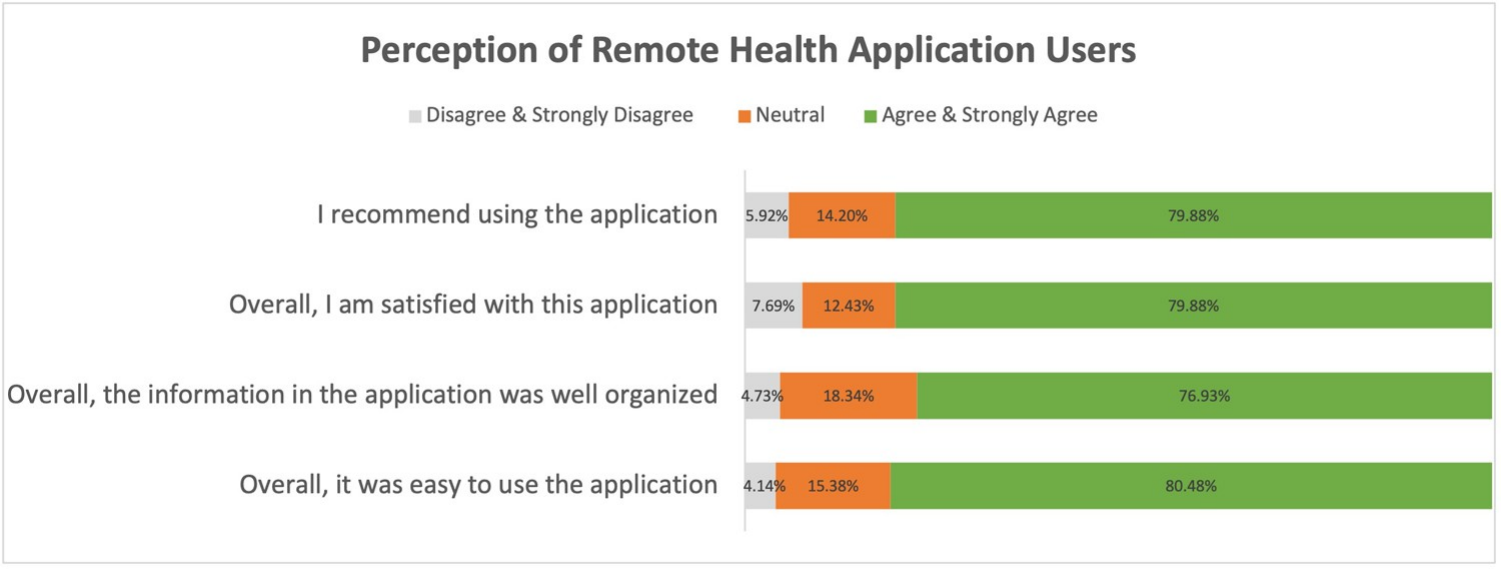
Perception of remote health application users.

Patients’ agreement with the quality of the mobile application was assessed (**Fig 1**). Overall, the majority of the mHealth “Remote Care” application users agreed that they recommend using the mobile application for telemedicine consultation (79.88%), are satisfied with the application (79.88%), such that they found it well organized and were able to find the needed information (76.93%), and found it easy to use for telemedicine consultations (80.48%).

## 4. Discussion

### 4.1. Principle findings

This study aimed to explore patients’ satisfaction with mobile health applications to manage and conduct telemedicine consultations. We have further explored patient characteristics and factors that predict satisfaction with the telemedicine service. This study’s findings suggested that the use of mobile health applications is associated significantly with ease of appointment booking. Another statistically significant finding in this study was that the care provided through telemedicine is regarded to be of similar quality as in-person consultations. Furthermore, it was found that there was a preference for using telemedicine over the in-person consultations during the COVID-19 pandemic.

### 4.2. mHealth application use associated with greater satisfaction and ease of telemedicine appointment booking

There is increasing evidence of the many benefits of using mobile health applications to the overall patient experience [28]. Our first key finding suggests that the use of a mobile health application is associated with increased ease of booking telemedicine appointments. In this study, patients who used the mobile application were almost 3 times more likely to find booking a telemedicine appointment easy compared to patients who used the traditional method for booking telemedicine appointments, which happens through the hospital’s operator system. This comes as no surprise as the advent of mHealth is to improve availability, accessibility, and communication in healthcare [29]. Booking appointments electronically or through the application has the major benefit of saving the patient a lot of time. In a study done by Zanaboni et al., it was shown that booking appointments using electronic resources proved to be more time efficient than conventional approaches [30]. Additionally, 80.48% of the patients in this study reported that the application as a whole was easy to use. This opens many doors to the possible utilities that can be used in this application other than booking and attending telemedicine appointments. One recommendation is for hospitals to implement a unified mobile health application that can offer telemedicine services in addition to other mHealth applications such as mobile sensors to monitor vital signs such as body temperature, heart rate, respiratory rate and blood pressure, and detect other disorders such as skin diseases [31], cardiovascular diseases [32,33], and a wide range of other diseases [34–38]. Hence, this key finding emphasizes the need to further encourage the usage of mobile health applications to improve healthcare access.

### 4.3. Care provided via telemedicine mobile applications is as good as in-person consultations

The second key finding of the paper is that telemedicine consults and in-person consultation were deemed equal in quality. The adjusted multivariate model further deduced that the mobile application users were almost twice as likely to agree that telemedicine consults provided the same quality of care as traditional in-person consultations. Despite the technical errors reported by the users in the study, they were satisfied with the care received. This has been supported by advancements in technology that enable patients to effectively self-assess and report findings to their physicians from the comfort of their homes. Remote patient monitoring (RMR), or telemonitoring, is the use of wearables, peripherals and telemedicine to monitor biometrics outside of a clinical setting, allowing clinicians to develop insights that allow for recommendations for the improvement of the individual’s health [39]. There is a multitude of use cases for RMR including chronic conditions such as diabetes and chronic obstructive pulmonary disease as well as for ageing populations and children with autism [40]. A pilot study by Kagiyama et al studied the validity of self-assessed vital signs in patients hospitalized with suspected or confirmed COVID-19. Patients were first successfully taught to use the devices to take their measurements via a 10-minute lecture. This study found that vital signs uploaded by the patients were in alignment with the measurements taken by healthcare providers for all parameters. These parameters included diastolic and systolic blood pressure, heart rate, peripheral oxygen saturation, body temperature, and respiratory rate [41]. Telemedicine has made further strides with the development of advanced mobile applications for chronically ill patients built with more assistance and behavioral change models that can increase patient satisfaction [42]. Providing accessible mHealth solutions for both patients and physicians has enhanced healthcare services and helped shift the practice to a more patient-centric model for self-management in cases of chronic conditions [43]. In line with this study’s findings, telemonitoring chronic diseases has managed at least equal quality of care as in-person visits, with less travel and waiting times and lower risk of hospital acquired infection, and ultimately highlighting telemedicine, remote patient monitoring, and associated patient applications as valuable tools and viable options for delivering a high quality of care to patients.

### 4.4. Preference for telemedicine over in-person visits during COVID-19

Since the start of the COVID-19 pandemic, there has been a trend of patients either delaying or avoiding seeking non-COVID related medical care due to concerns related to the COVID-19 virus, with this finding found to be more common in those with disabilities and those with two or more underlying conditions [44]. This provided a chance for telemedicine to bridge that gap, such that a study by McKinsey & Company found that within the first month of the pandemic, in April 2020, there had been a surge of 78 times more telemedicine users than in February 2020 [9]. This is supported by our study’s third key finding, that patients preferred telemedicine consults to in-person visits during the COVID-19 pandemic, with “Remote Care” users almost twice as likely to seek medical care through virtual consults as opposed to non-users. Such findings can be explained by considering that people with chronic conditions such as diabetes mellitus (DM) are at higher risk for COVID-19 infection and intensive care unit admission [45], and are therefore more likely to prefer remote consultation to avoid iatrogenic COVID-19 exposure [46]. Similarly, telemonitoring for chronically ill patients is necessary to reduce the risk of transmission and reduce morbidity and mortality. During the pandemic, chronically ill patients were also more likely to be anxious about being unable to visit their physician and have the same patient-physician experience [47,48]. However, telemedicine has bridged the gap with a virtual patient-physician communication experience and has thus reduced the ramping fear and anxiety associated with the hospital visit during the COVID-19 pandemic [42,47,48]. Furthermore, a study by Lin et al found that evaluating suspected COVID-19 patients using telemedicine has significantly helped reduce the risk of infection for physicians working in the emergency department and has played a role in infection control in The National Taiwan University Hospital [49].

### 4.5. Strenghts and limitations

This study has several strengths that may have significant implications for healthcare organizations. To the best of our knowledge, this study is the first to explore the effect of using a mobile health application in a telemedicine setting in Abu Dhabi. This study compared telemedicine consultations using a mobile health application with other conventional methods and measured the difference in patient satisfaction, which is useful for healthcare providers that are considering to start implementing mobile health applications. Along with these strengths lie several limitations. Firstly, this was a survey based cross-sectional study capturing a single point of time, with no longitudinal assessment of using mobile health applications for telemedicine after a long period of time. This limitation is further amplified by the fact that this study was conducted during the peak of the COVID-19 pandemic, with little to no information on how these results would translate in post COVID-19 times. Secondly, the data in this study was self-reported and is subject to recall bias, possibly skewing the results. Furthermore, as this survey was self-administered, it bears the risk of containing self-selection bias, with patients who favored the mobile health application being more motivated to take the survey. In addition, this study did not measure patient satisfaction with the other services of the mobile health application, which can influence the patients’ attitudes towards the application. Finally, our results could have been subjected to selection bias, since enrollment was based on volunteering, it is plausible that those who favored telemedicine or those who are proficient in the use of technology were more motivated to participate in this study.

## 5. Conclusion

Even though telemedicine acceptance and efficacy are widely documented in literature, little to no research has been done to prove its efficacy in a mobile health application setting. The explosive growth of mobile technologies paved the way for mHealth to prosper, and there is increasing evidence that suggests mHealth improves patient lives. Our study findings support that using a mobile health application is associated with increased ease of booking telemedicine appointments. This finding may be helpful for healthcare providers to create a unified mobile health application to improve patient care. In addition to making booking appointments easier, unified mobile health applications may also incorporate more technologies and applications such as mobile sensors that detect various diseases. This study also supported the finding that the care via telemedicine has the same quality as the hospital physical consultations, and demonstrated that patients preferred telemedicine consults over in-person visits during the COVID-19 pandemic. In addition to the positive findings for patients overall, these findings may be helpful for chronically ill patients, as they can manage their disease without having to visit a hospital and still get an equal quality of care, all with the benefit of having less travel and waiting times, and a decreased risk of acquired infection for both the patient and the physician.

## Supporting information

S1 Dataset (XLSX)
